# Supplementary material for: The role of loneliness in the development of depressive symptoms among partnered dementia caregivers: Evidence from the English Longitudinal Study of Aging
Source: Eur Psychiatry. 2021 Mar 26;64(1):e28. doi: 10.1192/j.eurpsy.2021.20 (PMC8080187; doi:10.1192/j.eurpsy.2021.20)
Supplement: Supplementary file 1 [file S0924933821000201sup001.docx]

**The role of loneliness in the development of depressive symptoms among partnered dementia caregivers: Evidence from the English Longitudinal Study of Ageing**

J. P. Saadi*^1^, E. Carr*^2,3^, M. Fleischmann^4^, E. Murray^2^, J. Head^2^, A. Steptoe^1,2^, R. A. Hackett^1,5^, B. Xue^2^, D. Cadar^1^

^1^ Department of Behavioural Science and Health, University College London, UK

^2^ Department of Epidemiology and Public Health, University College London, London, UK

^3^ Department of Biostatistics and Health Informatics, Institute of Psychiatry, Psychology & Neuroscience, King’s College London, London, UK

^4^ Faculty of Science, Methodology and Applied Biostatistics, University of Amsterdam, the Netherlands

^5^ Department of Psychology, King’s College London, London, UK

* Joint first authors. Both authors contributed equally to this work.

Address correspondence to:

Dorina Cadar

Department of Behavioural Science and Health

University College London, UK.

1-19 Torrington Place

WC1E 7HB

E-mail: [d.cadar@ucl.ac.uk](mailto:d.cadar@ucl.ac.uk)

**Keywords:** Loneliness, depression, and dementia care

**Abstract**

**Background.** Depressive symptoms are highly prevalent among partnered dementia caregivers, but the mechanisms are unclear. This study examined the mediating role of loneliness in the association between dementia care on subsequent depressive symptoms.

**Method.** Prospective data from partnered caregivers were drawn from the English Longitudinal Study of Ageing. The sample consisted of 4,672 partnered adults aged 50-70 living in England and Wales, followed up between 2006-07 and 2014-15. Caregiving was assessed across waves 3 (2006-07), 4 (2008-09) and 5 (2010-11), loneliness at wave 6 (2012-13), and subsequent depressive symptoms at wave 7 (2014-15). Multivariable logistic regression models were used to assess the association between dementia and other types of caregiving (e.g. diabetes, CHD, cancer, and stroke) on depressive symptoms. Binary mediation analysis was used to estimate the indirect effects of caregiving on depressive symptoms via loneliness.

**Results.** Care for a partner with dementia was associated with higher odds of depressive symptoms at follow-up compared to those not caring for a partner at all (Odds Ratio (OR)=2.6, 95% Confidence Intervals (CI): 1.4, 5.1). This association was partially mediated by loneliness (34%). Care for a partner with other conditions was also associated with higher odds of depressive symptoms compared to non-caregiving partners (OR=1.7, 95% CI: 1.2, 2.5); but there was no evidence of an indirect pathway via loneliness.

**Conclusion.** Loneliness represents an important contributor to the relationship between dementia caregiving and subsequent depressive symptoms; therefore, interventions to reduce loneliness among partnered dementia caregivers should be considered. **Keywords:** dementia caregiving, dementia, loneliness, depressive symptoms

**Key points**

- Partner dementia caregiving was associated with increased odds of depressive symptoms at follow-up.
- The relationship between dementia caregiving and depressive symptoms was partially mediated by caregiver loneliness.
- These findings highlight the importance of loneliness associated with caring for a partner with dementia on the subsequent mental health of carers, emphasising the need to consider the role of loneliness in interventions aimed to support dementia caregivers.

# Introduction

Caring for an individual with dementia has been associated with poor mental health outcomes, but the mechanisms are less understood [1, 2]. Given the increasing number of dementia caregivers globally and potential negative impact on their mental wellbeing [3], identifying modifiable mechanisms that can be targeted through cost-effective interventions is important [4].

A meta-analysis of 84 studies found that caregivers were more likely to report depressive symptoms compared to non-caregivers [5]. The incidence of depressive symptoms depends on the characteristics of both the caregiver and recipient [6]. Recipient characteristics such as younger age (i.e. <65 years old compared to ≥ 85), lower education, Hispanic ancestry, higher levels of disability, and the presence of challenging behaviours have all been associated with higher depressive symptoms among caregivers [6]. Being the spouse of a care recipient (especially the wife) is also a risk factor for depression compared to other types of caregiver roles, such as caring for grandchildren [6].

Caring for an individual with dementia involves both physical and neurological symptomatology, as well as behavioural and cognitive impairments, which may explain why dementia care incurs a greater burden compared to caring for an individual with only physical (but not neural) impairments [7]. The demands of dementia care could also lead to loneliness among the caregiver, and it is well documented that loneliness is linked with reduced mental wellbeing as well as physical morbidity and increased mortality [8]. A UK-based prevalence study indicated that over two-thirds of dementia caregivers reported feeling lonely [9]. The study found that greater social isolation and caregiver stress were associated with higher loneliness, while caregiver-recipient relationship quality was protective.

The relationship between loneliness and depression is well established, but few studies have examined this in relation to dementia caregiving. Loneliness was a strong predictor of depressive symptoms among 242 spousal dementia caregivers in the USA [10], and a cross-sectional study of 49 spousal dementia caregivers found loneliness to explain 49% of the variance depressive symptoms [11]. Caregiver loneliness might increase in parallel with the increasing severity of dementia, which could elevate the risk of adverse mental health among caregivers over time. However, most previous studies have relied on cross-sectional data and have not addressed potential mechanistic relationships between caregiving and depressive symptoms.

This study considered whether the higher risk of depressive symptoms among those caring for a partner with dementia (compared to non-caregiving partners) could be explained by loneliness. We tested the following hypotheses:

1. Individuals providing care for a partner with dementia (‘dementia caregiving’) would have increased odds of depressive symptoms at follow-up compared to individuals not providing care for their partner (irrespective of partner dementia diagnosis).
2. Individuals caring for a partner with dementia would report higher levels of loneliness compared to other partnered caregivers.
3. Loneliness would mediate the relationship between dementia caregiving and subsequent depressive symptoms.

## Method

### Design, setting and participants

Data were drawn from the English Longitudinal Study of Ageing (ELSA), an ongoing nationally representative sample of approximately 11,000 individuals aged 50 and over living in England [12]. Participants were initially recruited in 2002-03. Refreshment samples of new participants were recruited in 2006-07, 2008-09, 2010-11, 2012-13, and 2014-15. All waves took place biennially consisting primarily of self-completed questionnaires and face-to-face interviews. Only waves 3-7 were used in this analysis due to the lack of caregiving data prior to wave 3. We included in our analysis refreshment samples introduced at waves 3 and 4. We selected 10,813 individuals who had provided care to their partner between wave 3 (2006-07) and wave 5 (2010-11) and had available data on loneliness at wave 6 (2012-13) and depressive symptoms at wave 7 (2014-15). Participants were only eligible if they had a partner.

**Measurements**

*Caregiving*. Caregiving status was assessed by self-report questions at waves 3, 4, and 5 where participants were asked: “Did you look after anyone in the last week (including your partner or other people in your household)?” Participants responding “No” were classified as ‘non-caregiving partners’. Those responding “Yes” were then asked what their relation to the care recipient was (e.g. partner, child). Those not providing care to a partner were excluded from the final analytic sample. Individuals who provided care for a partner at least once across waves 3, 4, or 5 were classified as a caregiver. Functional impairments were assessed at waves 3, 4 and 5, by asking whether household members required support with Activities of Daily Living (ADLs; bathing or showering, walking across a room, dressing, getting in/out of bed, eating, using the toilet) or Instrumental Activities of Daily Living (IADLs; preparing a hot meal, doing house/garden work, using a map in unknown places, grocery shopping, taking medication, making telephone calls, managing money) [13]. Dementia status was assessed based on self-reported physician-confirmed diagnoses of dementia at waves 3, 4 and 5.

Based on this information, we derived a four-level caregiving measure: 0=‘Non-caregiving partners’, the reference category; 1=‘Care for partner with dementia’; 2=‘Care for partner with functional impairments (but no diagnosis of dementia)’; and 3=‘Care for partner with other conditions’. For mediation analyses, we created binary dummy variables representing each caregiving category.

*Depressive symptoms* Self-reported depressive symptoms were assessed at wave 7 (2014/15) using the eight-item version of the Center for Epidemiologic Studies Depression Scale (CES-D) [15], see Table S1. This measure had good internal reliability across waves (α=0.84) and comparable psychometric properties to the full 20-item scale [16, 17]. All items were coded as “Yes” or “No”. The item addressing loneliness was removed to ensure that this item did not overlap with the mediating effect of loneliness [18]. The remaining seven items were summed and dichotomised, such that participants with scores of ≥3 were considered with elevated depressive symptoms) [19-20].

*Potential mediator*. Loneliness was measured using the three-item short form of the revised University of California, Los Angeles (UCLA) Loneliness Scale, which has been shown to have acceptable internal reliability (α=0.78) [21]. Three questions assessed the frequency that an individual had felt a lack of companionship, left out, or isolated from others, over the past week. Answers were scored on a three-point scale ranging from 1 (“Hardly ever or never”) to 3 (“Often”) and summed to create a continuous total score ranging 3-9. Higher scores indicated increased loneliness [22].

*Covariates.* Analyses were adjusted for covariates measured at the first non-missing interview between waves 3 and 5. Adjustments were made for gender, age, household wealth, marital status, ethnicity, highest educational qualification, employment status, presence of limiting long-standing illness, and poor self-rated health.

To reduce possible confounding, all fully adjusted models were estimated with adjustment for baseline depressive symptoms (CES-D score of ≥3 at wave 3; or wave 4, if missing at wave 3). Please see supplementary material for more information on how and why these covariates were chosen/derived.

## *Statistical analysis*

χ^2^ and Kruskal-Wallis tests were used to investigate baseline demographic differences between the four caregiving categories, as well as differences in depressive symptoms at follow-up. The association between caregiving and depressive symptoms was assessed using logistic regression models. Three models were estimated (1) unadjusted, (2) adjusted for age, gender, education level, household wealth, marital status, ethnicity, employment status and (3) additionally adjusted for the presence of long-standing limiting illness, self-rated health, and baseline depressive symptoms. All models were estimated in the overall sample and separately for men and women. We reported the Odds Ratios (OR) and 95% Confidence Intervals (CI).

Mediation was assessed by fitting a series of linear regression models for continuous variables (loneliness) and logistic regression models for binary variables (depressive symptoms) using the ‘medeff’ statistical package in Stata 14 [23, 24]. Due to differences in sample size across these groups, the sample sizes for assessing mediation vary slightly. The manuscript was written following STROBE guidelines [25], see Table S2.

### Sensitivity analyses

To reduce possible confounding, all models were reestimated after excluding participants meeting criteria for clinically significant depressive symptoms at baseline (7-items CES-D score of ≥3 at wave 3 or wave 4 if missing at wave 3). A second sensitivity analysis was conducted on the main sample using the 8-item CES-D with a threshold of ≥ 4 at wave 3 or wave 4, if missing at wave 3, instead of the ≥ 3 threshold used in the main analysis

## Results

### Sample characteristics

The analytical sample included 4,672 participants after removal of those with missing information on caregiving status (*n*=1), covariates (*n*=739), mediators (*n*=74) or depressive symptoms (*n*=928) (Figure 1).

<Figure 1>

Compared to the main sample, excluded participants were older (64.8 vs. 61.9 years), more likely to be female (59% vs. 51%), similar in terms of non-white ethnicity (4% vs. 3%), had a higher incidence of long-standing limiting illnesses (37% vs. 26%), were less likely to have completed any formal education (minimum of O-level award or junior/middle high school diploma) (67% vs. 80%), were more likely to be in the lowest quintile of household wealth (25% vs. 8%) and were less likely to be employed (35% vs. 46%).

Table 1 presents the demographic characteristics of the analytical sample stratified by caregiving status. Compared to non-caregiving partners, those caring for a partner were more likely to be female than male (59% vs. 51%), older (63.5 vs. 61.8 years), to be in the lowest quintile of household wealth (18% vs. 9%), less likely to be employed (30% vs. 48%) and more likely to report long-standing limiting illnesses (35% vs. 25%). However, non-caregivers at baseline were less likely to report depressive symptoms (9% vs. 13%) and slightly lower levels of loneliness (mean of 3.8 vs. 4.0) compared to caregivers. All differences were statistically significant at p<0.005 except for differences in ethnicity (*p*=0.413).

Dementia caregivers tended to be older (68.0 vs. 61.8 years; *p*<0.001), female (69% vs. 51%; p<0.007) and unemployed (10% vs. 48%; *p*<0.001), compared to partners not providing any care. The proportion of dementia caregivers reporting depressive symptoms was higher compared to non-caregiving partners (at baseline: 21% vs. 9%; *p*<0.001; at follow-up: 27% vs. 13%; *p*<0.001) and when compared to those caring for a partner with functional impairments (baseline: 21% vs. 11%; p=0.03; follow-up: 38% vs. 20%; p<0.01). Dementia caregivers were also more likely to report depressive symptoms at follow-up (27% vs. 22%; p<0.01) compared to those caring for a partner with other conditions, but this difference did not reach statistical significance at baseline (21% vs. 13%; p=0.09). Dementia caregivers were more likely to report long-standing limiting illnesses at baseline (44% vs. 25%; p<0.005).

<Table 1>

### The relationship between caregiving status and subsequent depressive symptoms at follow-up

In the overall sample (*n*=4,672), individuals caring for their partner had higher odds of developing depressive symptoms compared with those not caring for their partners. This was especially the case among those providing care to a partner with dementia (Table 2). In unadjusted analyses, caregiving for a partner with dementia (OR=3.99, 95% CI: 2.21, 7.20), functional impairments (OR=1.68, 95% CI: 1.16, 2.45), or other conditions (OR=1.82, 95% CI: 1.28, 2.60) was associated with increased levels of depressive symptoms. This relationship was attenuated after full adjustment and remained statistically significant only for those caring for a partner with dementia or other conditions. Preliminary analyses found no gender differences between various types of caregiving within those with depressive symptoms at follow-up (*χ^2^* = 5.73, *p* = 0.13).

<Table 2>

### The mediating role of loneliness (wave 6) on the association between caregiving (waves 3-5) and depressive symptoms (wave 7)

Caring for a partner with dementia was positively associated with loneliness (*β*=0.31, 95% CI: 0.15, 0.47), and loneliness was positively associated with depressive symptoms at follow-up (OR = 1.11, 95% CI: 0.92, 1.30). Moreover, caring for a partner with dementia was indirectly related to depressive symptoms via loneliness (*β*=0.04, Bias corrected (Bc) CI: 0.02, 0.07), which explained 34% of the total effect.

<Figure 2>

Caring for a partner with other conditions was not significantly associated with loneliness (*β*=-0.004, 95% CI: -0.07, 0.06), and although loneliness was positively related to depressive symptoms (OR=1.13, 95% CI: 0.94, 1.32), there was no evidence of an indirect effect via loneliness (*β*=-0.001, Bc CI: -0.008, 0.008). Since caring for a partner with functional impairments was not associated with depressive symptoms after full adjustment (i.e. no ‘Total effect’), this model was not tested for mediation.

<Figure 3>

### Sensitivity analyses

We conducted two sensitivity analyses. Firstly, we excluded all participants who met the criteria for baseline depressive symptoms (*N*=423), see Tables S3. In the second analysis, we reestimated all models using the original CES-D scale (with a cut-off of ≥4), see Tables S4. The new results did not substantially alter the strength of the initial associations found between caregiving types and depressive symptoms at follow-up compared to our main analyses.

Moreover, the indirect effect of caregiving on depressive symptoms via loneliness remained statistically significant among dementia caregivers and non-significant among those in the “other care” category in both sensitivity analyses (see Figures S1 to S4).

## Discussion

We found that caring for a partner with dementia was associated with higher odds of depressive symptoms at follow-up compared to non-caregiving partners and that loneliness significantly mediated this association. A similar association was observed for those caring for other conditions than dementia, but the increased odds of depressive symptoms was lower than for those caring for a partner with dementia. We did not find evidence to suggest loneliness mediates the association between care for a partner with other conditions and subsequent depressive symptoms.

Consistent with our first hypothesis, the odds of depressive symptoms were higher among dementia caregivers when compared to non-caregiving partners. This is in line with previous research [5, 6] and may reflect the caregiving burden associated with increased psychological and mental demands, as well as the physical and behavioural challenges that can present in patients with dementia and the strain these can place on the couple [7]. However, we did not find evidence of gender differences in the relationships between caregiving and depressive symptoms, consistent with one other previous study [28]. Caring for individuals with dementia requires ongoing care that may impose withdrawal from paid work, which is another determinant of poor mental health [26, 27]. While some partners may embrace the caregiving role, some report feelings of ‘role captivity’ which is likely to feed into the development of depressive symptoms [29]. Role captivity refers to a sense of being trapped in a specific social role which limits the individual’s freedom. This might partly be responsible for feelings of loneliness and depressive symptomatology.

Our findings might support the idea that caring for a partner with both mental and functional impairments (i.e. dementia) is more strenuous than caring for a partner with only functional impairments or physical disability [7]. Although we did not make an active comparison between these groups, this was inferred from the larger odds ratio in the dementia care group compared to the functional impairments group. Notably, caring for a partner with only physical, rather than both mental and physical impairments as in the case of dementia, was not found to be associated with subsequent higher odds of depressive symptoms. This is thought-provoking because it suggests that caring for partners with physical impairments only is not necessarily linked to poorer mental health. It is also possible that greater support is available to carers for partners with only functional impairments, or that those caring for a partner with dementia have less time and opportunities for social support, and therefore more vulnerable to an increased risk of depressive symptoms. Ultimately, it may be that it is the addition of psychological/behavioural impairments that tips the association towards significance.

Consistent with our second hypothesis, loneliness was highest among dementia caregivers compared to non-caregiving partners. A possible explanation for this is the shift from partnership to the caregiver-patient role, and thus the loss of a previously meaningful relationship. This is consistent with qualitative findings describing the caregiving experience as one of ‘relational deprivation’ [30]. The burden of caregiving activities may increase loneliness by reducing the opportunities to engage with a wider social circle [10]. Similarly, the progressive deterioration in cognitive functioning among those with dementia often also results in personality changes, that may precipitate a significant emotional loss in the form of anticipatory grief [31-33]. Both of these factors may contribute to increased loneliness.

Consistent with our third hypothesis, we found evidence of indirect effects of dementia caregiving on depressive symptoms via loneliness, highlighting the importance of considering loneliness when supporting such individuals. Interestingly, there was a direct effect of caring for other conditions on depressive symptoms; however, we did not find any indirect effect via loneliness. This suggests that policies and/or therapeutic interventions need to be tailored to the specific needs of the couple. Future research could attempt to uncover what are the specific mediating factors of the ‘other care’ category.

### Strengths and limitations

Our analysis represents a longitudinal investigation of dementia caregivers living in their community and their subsequent level of loneliness and mental health, using a representative sample of the English population. This is a significant benefit since many studies of caregivers are based on the recruitment of participants who have self-identified as distressed caregivers, a factor likely to inflate the findings and reduce ecological validity [34]. We also used a superior design compared to past studies where comparison groups have contained both partnered and un-partnered non-caregivers. Being married or having a partner is usually protective of mental health [35], but previous studies may have underestimated the effect of caregiving on depressive symptoms. Furthermore, we used a prospective longitudinal data that allowed us to test potential causal inferences regarding the impact of partner caregiving on subsequent loneliness and depressive symptoms.

However, several potential limitations have been identified. Firstly, caregiving was based on a self-reported measure, and no consideration was given to the intensity of care provided or the duration of the caregiving role (due to sample size limitations). Moreover, our measurement of caregiving did not allow variations in caregiving intensity or transitions in/out of a caregiving role, which previous studies have identified as important for mental health outcomes [36]. By not considering the potential transitions in caring roles may have introduced some bias into our findings. However, we assumed that once an individual becomes a carer for a partner with dementia, their role as caregiver is likely to continue for some time (either until the partner dies, or becomes institutionalised). Furthermore, we assumed that even if caregivers transitioned out of the role between waves 3 and 5, the influence on their mental health would be expected to continue to some degree. This would be in line with the relational deprivation hypothesis, which suggests that it is partly the meaning attributed to the caregiving role (i.e. that it acts as a kind of loss of the partner once known) that mediates depressive symptoms (rather than solely the requirements of the role itself).

Secondly, it is also worth noting that although dementia diagnoses were made by a physician, it was still a self-reported physician-based diagnosis reported by either the participant themselves or their carers. This, however, may raise some questions around the reliability of the diagnosis for these analyses, although it is unlikely that such reports will be false positives. In fact, it is estimated that dementia remains undetected in almost 30 to 50% of primary care patients in the UK [37]. Furthermore, it is possible that care-recipients relevant to the “functional impairments” category had dementia during the exposure period but had not yet received a diagnosis by a physician. This may have attenuated differences between each group.

Thirdly, we had no means of knowing whether caregivers were receiving psychological support and the number of individuals with dementia was small, both of which could have affected the results. Similarly, excluding participants with missing data could have diluted the reported effect sizes. For example, based on their characteristics (see results section), excluded participants might be more at risk of depressive symptoms compared to those without missing data. While the reasons for attrition among ELSA participants have been previously explored, it is unclear whether the caregiving role had any influence on this attrition, and what the impact on the results was [36].

Fourthly, to maximise the use of available data, our measurement of caregiving captured caring roles at either wave 3, 4, or 5. We took this approach because if an individual entered a caregiving role at waves 4 or 5, we wanted to avoid classifying them as “Not caring” based on wave 3 alone. As discussed above, a caregiving role at any single wave may still be associated with subsequent depressive symptoms. This measurement of caregiving across multiple waves introduced a further limitation whereby for a small minority (less than 5% of the analytical sample) caregiving status and covariates were measured at different waves. However, for most of our covariates with the exception of age, we did not expect a real change over time.

Finally, although we found evidence of an indirect effect of dementia caregiving on depressive symptoms via loneliness, it was unclear whether it was due to the caregiving itself or the death of the care-recipient, for example. Having said that, the specific causes of loneliness are likely to vary between individuals, and this would be explored as part of any therapeutic intervention.

**Conclusion**

Our findings indicate that partner dementia caregiving is indirectly associated with depressive symptoms via feelings of loneliness. The mental health of dementia caregivers is arguably an essential psychiatric priority, given that it could seriously influence the quality of care provided to their partner, and thus impact the rate of care-recipient institutionalisation [34, 39].

**ACKNOWLEDGEMENTS**

The English Longitudinal Study of Ageing is funded by the National Institute on Aging (Grant RO1AG017644) and by a consortium of UK government departments coordinated by the Economic and Social Research Council (ESRC) and the National Institute for Health Research. The English Longitudinal Study of Ageing (ELSA) was developed by a team of researchers based at University College London, the Institute for Fiscal Studies and the National Centre for Social Research. DC is funded by the National Institute on Aging (grant RO1AG017644) and ESRC (grants ES/T012091/1 & ES/S013830/1). EC is funded by the National Institute for Health Research (NIHR) Biomedical Research Centre at South London and Maudsley NHS Foundation Trust and King’s College London. The views expressed are those of the author and not necessarily those of the NHS, the NIHR or the Department of Health and Social Care.

###### **Sponsor’s role**

The views expressed are those of the authors and not of the funding agencies.

**Conflicts of interest**

On behalf of all co-authors, we have no conflict of interest to declare.

**Data Availability Statement**

The ELSA data containing the individual items and the derived scores have been made available via UK Data Service (<http://www.esds.ac.uk/longitudinal/access/elsa>), and the study number is 5050 (<http://doi.org/10.5255/UKDA-SN-8502-2)>.

**References**

[1] Sallim AB, Sayampanathan AA, Cuttilan A, Ho R. Prevalence of mental health disorders among caregivers of patients with Alzheimer's disease. J Am Med Dir Assoc. 2015; 16: 1034-41. https://doi.org/10.1016/j.jamda.2015.09.007

[2] Sörensen S, Conwell Y. Issues in dementia caregiving: Effects on mental and physical health, intervention strategies, and research needs. Am J Geriatr Psychiatry. 2013;19: 491-6. https://doi.org/10.1097/JGP.0b013e31821c0e6e

[3] Alzheimer’s Association. Alzheimer’s Disease Facts and Figures. Alzheimer's Dement. 2017; 13: 325-73. https://doi.org/10.1002/alz.12068

[4] Hoff A. Current and Future Challenges of Family Care in the UK: future of an ageing population. https://www.gov.uk/government/publications/future-of-ageing-family-care-in-the-uk; 2015 [accessed 18 January 2020]

[5] Pinquart M, Sörensen S. Differences between caregivers and noncaregivers in psychological health and physical health: a meta-analysis. Psychol Aging. 2003; 18: 250-67. https://doi.org/10.1037/0882-7974.18.2.250

[6] Covinsky KE, Newcomer R, Fox P, et al. Patient and caregiver characteristics associated with depression in caregivers of patients with dementia. J Gen Intern Med. 2003; 18: 1006-14. https://doi.org/10.1111/j.1525-1497.2003.30103.x

[7] Ory MG, Hoffman RR, Yee JL, Tennstedt S, Schulz R. Prevalence and impact of caregiving: a detailed comparison between dementia and nondementia caregivers. Gerontologist. 1999; 39: 177-85. https://doi.org/10.1093/geront/39.2.177

[8] Hawkley LC, Cacioppo JT. Loneliness matters: a theoretical and empirical review of consequences and mechanisms. Ann Behav Med. 2010; 40: 218-27. https://doi.org/10.1007/s12160-010-9210-8

[9] Victor CR, Rippon I, Quinn C, Nelis SM, Martyr A, Hart N et al. The prevalence and predictors of loneliness in caregivers of people with dementia: findings from the IDEAL programme. Aging Ment Health. 2020; 1-7. https://doi.org/10.1080/13607863.2020.1753014

[10] Beeson RA, Horton-Deutsch S, Farran C, Neundorfer M. Loneliness and depression in caregivers of persons with Alzheimer's disease or related disorders. Issues Ment Health N. 2000; 21: 779-806. https://doi.org/10.1080/016128400750044279

[11] Beeson RA. Loneliness and depression in spousal caregivers of those with Alzheimer's disease versus non-caregiving spouses. Arch Psychiatr Nurs. 2003; 17: 135-43. https://doi.org/10.1016/S0883-9417(03)00057-8

[12] Steptoe A, Breeze E, Banks J, Nazroo J. Cohort Profile: The English Longitudinal Study of Ageing. Int J Epidemiol. 2013; 42: 1640-8. https://doi.org/10.1093/ije/dys168

[13] Torres JL, Lima-Costa MF, Marmot M, de Oliveira C. Wealth and disability in later life: The English Longitudinal Study of Ageing (ELSA). PLoS One. 2016; 11: e0166825. https://10.0.5.91/journal.pone.0166825

[14] Bradford A, Kunik ME, Schulz P, Williams SP, Singh H. Missed and delayed diagnosis of dementia in primary care: prevalence and contributing factors. Alzheimer Dis Assoc Disord. 2009; 23: 306-14. https://doi.org/10.1097/WAD.0b013e3181a6bebc

[15] Radloff L. The CES-D scale: A self-report depression scale for research in the general population. Appl Psychol Meas. 1977; 1: 385-401. https://doi.org/10.1177/014662167700100306

[16] Rafnsson SB, Shankar A, Steptoe A. Informal caregiving transitions, subjective well-being and depressed mood: Findings from the English Longitudinal Study of Ageing. Aging Ment Health. 2017; 21: 104-12. https://doi.org/10.1080/13607863.2015.1088510

[17] Turvey CL, Wallace RB, Herzog R. A revised CES-D measure of depressive symptoms and a DSM-based measure of major depressive episodes in the elderly. Int Psychogeriatr. 1999; 11: 139-48. https://doi.org/10.1017/s1041610299005694

[18] Cacioppo JT, Hawkley LC, Thisted RA. Perceived social isolation makes me sad: 5-year cross-lagged analyses of loneliness and depressive symptomatology in the Chicago Health, Aging, and Social Relations Study. Psychol Aging. 2010; 25: 453-63. <https://doi.org/10.1037/a0017216>

[19] Daly M. The relationship of C-reactive protein to obesity-related depressive symptoms: a longitudinal study. Obesity (Silver Spring). 2013 Feb;21(2):248-50. doi: 10.1002/oby.20051. PMID: 23404927.

[20] White J, Zaninotto P, Walters K, Kivimäki M, Demakakos P, Biddulph J, Kumari M, De Oliveira C, Gallacher J, Batty GD. Duration of depressive symptoms and mortality risk: the English Longitudinal Study of Ageing (ELSA). Br J Psychiatry. 2016 Apr;208(4):337-42. doi: 10.1192/bjp.bp.114.155333. Epub 2016 Jan 21. PMID: 26795425; PMCID: PMC4816969.

[21] Hughes ME, Waite LJ, Hawkley LC, Cacioppo JT. A Short Scale for Measuring Loneliness in Large Surveys: Results From Two Population-Based Studies. Res Aging. 2004; 26: 655-72. https://doi.org/10.1177/0164027504268574

[22] Shankar A, Hamer M, McMunn A, Steptoe A. Social isolation and loneliness: relationships with cognitive function during 4 years of follow-up in the English Longitudinal Study of Ageing. Psychosom Med. 2013; 75: 161-70. https://doi.org/10.1097/PSY.0b013e31827f09cd

[23] Hicks R, Tingley D. Causal mediation analysis. The Stata Journal. 2011; 11: 605–19.

[24] StataCorp. 2014. Statistic Software: Release 7.0. College Station, TX: Stata Corporation.

[25] von Elm E, Altman DG, Egger M, Pocock SJ, Gøtzsche PC, Vandenbroucke JP; STROBE Initiative.The Strengthening the Reporting of Observational Studies in Epidemiology (STROBE)statement: guidelines for reporting observational studies. Lancet. 2007 Oct 20;370(9596):1453-7. PMID: 18064739

[26] Sefcik J, Petrovsky D, McPhillips MV, Hodgon N, Gitlin L. Financial strain among dementia informal caregivers. Innov Aging. 2018; 2: 76. https://doi.org/10.1093/geroni/igy023.290

[27] Quinn C, Clare L, Woods RT. The impact of motivations and meanings on the wellbeing of caregivers of people with dementia: a systematic review. Int Psychogeriatr. 2019; 22: 43-55. https://doi.org/10.1017/S1041610209990810

[28] Pöysti MM, Laakkonen ML, Strandberg T, et al. Gender differences in dementia spousal caregiving. Int J Alzheimers Dis. 2012; 162960. <https://doi:10.1155/2012/162960>

[29] Givens JL, Mezzacappa C, Heeren T, Yaffe K, Fredman L. Depressive symptoms among dementia caregivers: role of mediating factors. Am J Geriatr Psychiatry. 2014; 22: 481-88. https://doi.org/10.1016/j.jagp.2012.08.010

[30] Vasileiou K, Barnett J, Barreto M, Vines J, Atkinson M, Lawson S, Wilson M. Experiences of Loneliness Associated with Being an Informal Caregiver: A Qualitative Investigation. Front Psychol. 2017; 8: 585. https://doi: 10.3389/fpsyg.2017.00585

[31] Robins Wahlin TB, Byrne GJ. Personality changes in Alzheimer’s disease: a systematic review. Int J Geriatr Psychiatry. 2011; 26: 1019-29. https://doi.org/10.1002/gps.2655

[32] Hazan C, Shaver PR. Romantic love conceptualised as an attachment process. J Pers and Soc Psychol. 1987; 52: 511-524. https://doi.org/10.1037/0022-3514.52.3.511

[33] Blandin K, Pepin R. Dementia grief: A theoretical model of a unique grief experience. Dementia (London). 2015; 16: 67-78. https://doi.org/10.1177/1471301215581081

[34] Ask H, Langballe E, Holmen J, Selbæk G, Saltvedt I, Tambs K. Mental health and wellbeing in spouses of persons with dementia: the Nord-Trøndelag health study. BMC Public Health. 2014; 14: 1-12. <https://doi.org/10.1186/1471-2458-14-413>

[35] Akhtar-Danesh N, Landeen J. Relation between depression and sociodemographic factors. Int J Ment Health Syst. 2007; 1: 4. https://doi:10.1186/1752-4458-1-4

[36] Seltzer MM, Li WL. The dynamics of caregiving: transitions during a three-year prospective study. The Gerontol Soc Amer. 2000; 40: 165-78. <https://doi.org/10.1093/geront/40.2.165>

[37] Connolly A, Gaehl E, Martin H, Morris J, Purandare N. Underdiagnosis of dementia in primary care: variations in the observed prevalence and comparisons to the expected prevalence. Aging Ment Health 2011;15:978–84.

[38] Banks J, Muriel A, Smith JP. Attrition and health in ageing studies: Evidence from ELSA and HRS. Longit Life Course Stud. 2011; 2: 24376472. https://doi:10.14301/llcs.v2i2.115

[39] Coehlo DP, Hooker K, Bowman S. Institutional placement of persons with dementia: what predicts occurrence and timing? J Fam Nurs. 2007; 13: 253-77. https://doi.org/10.1177/1074840707300947

Table 1. Demographic characteristics of the analytic sample (*n*=4,672)

|  | | | **Non-caregiving partners**  **(*n*= 4,251)** | **Partner caregivers (*n*= 421)** | | | |
| --- | --- | --- | --- | --- | --- | --- | --- |
|  |  |  |  | All caregivers  (*n*= 421) | Care for partner with dementia  (*n* = 48)^a^ | Care for partner with functional impairments  (*n* = 178) | Other types of partner care  (*n*= 195) |
| *Baseline covariates* | | |  |  |  |  |  |
|  | Age (year), mean (*SD*) | | 61.8 (7.5) | 63.5 (8.0) | 68.0 (7.7) | 63.6 (8.1) | 62.3 (7.7) |
|  | Gender (female), *n* (*%*) | | 2,145 (50.5) | 248 (58.9) | 33 (68.8) | 91 (51.1) | 124 (63.6) |
|  | Ethnicity (White), *n* (*%*) | | 4,131 (97.3) | 412 (97.9) | 46 (95.8) | 176 (88.0) | 190 (97.4) |
|  | Household wealth, *n* (*%*) | |  |  |  |  |  |
|  |  | 1^st^ (Lowest) | 320 (7.5) | 74 (17.6) | 9 (18.8) | 40 (22.5) | 25 (12.8) |
|  |  | 2^nd^ | 631 (14.8) | 78 (18.5) | 9 (18.8) | 42 (23.6) | 27 (13.9) |
|  |  | 3^rd^ | 863 (20.3) | 72 (17.1) | 8 (16.7) | 27 (15.2) | 37 (19.0) |
|  |  | 4^th^ | 1,079 (25.4) | 103 (24.5) | 11 (22.9) | 38 (21.4) | 54 (27.7) |
|  |  | 5^th^ (Highest) | 1,358 (31.9) | 94 (22.3) | 11 (22.9) | 31 (17.4) | 52 (26.7) |
|  | Education level, *n* (*%*) | |  |  |  |  |  |
|  |  | No qualification (elementary school diploma) | 847 (19.9) | 100 (23.8) | 13 (27.1) | 48 (27.0) | 39 (20.0) |
|  |  | Up to GCE O-level (middle or junior high school diploma) | 1,010 (23.8) | 110 (26.1) | 10 (20.8) | 60 (33.7) | 40 (20.5) |
|  |  | Up to A-level/ Equivalent (high school or senior high school diploma) | 666 (15.7) | 67 (15.9) | 13 (27.1) | 22 (12.4) | 32 (16.4) |
|  |  | Lower than degree | 771 (18.1) | 68 (16.2) | 4 (8.3) | 25 (14.0) | 39 (20.0) |
|  |  | Degree (university undergraduate certificate) | 957 (22.5) | 76 (18.1) | 8 (16.7) | 23 (12.9) | 45 (23.1) |
|  | Employed, *n* (*%*) | | 2,036 (47.9) | 124 (29.5) | 5 (10.4) | 49 (16.3) | 70 (35.9) |
|  | Married, *n* (*%*) | | 3,949 (92.9) | 399 (94.8) | 46 (95.8) | 168 (94.4) | 185 (94.8) |
|  | Self-rated health (1-5), mean (*SD*) | | 2.57 (1.0) | 2.77 (1.1) | 2.83 (1.1) | 2.92 (1.2) | 2.61 (1.0) |
|  | Long-standing limiting illness, *n* (*%*) | | 1,075 (25.3) | 148 (35.2) | 21 (43.8) | 71 (39.9) | 56 (28.7) |
| *Mediator* | | |  |  |  |  |  |
|  | UCLA Loneliness scale score, *mean (SD)* | | 3.93 (1.4) | 4.31 (1.6) | 5.08 (1.7) | 4.43 (1.6) | 4.00 (1.4) |
| *Follow-up* | | |  |  |  |  |  |
|  | CES-D depressive symptoms scale score (≥3), *n (%)* | | 556 (13.1) | 96 (22.8) | 18 (37.5) | 36 (20.2) | 42 (21.5) |

*Note.* *SD* = standard deviation; *n* = sample size

Table 2. The odds ratios of depressive symptomatology (wave 7) among caregivers (waves 3-5) in the full analytic sample (*N*=4,672)

|  | **Full sample (n=4,672)** | | | **Males (n=2,279)** | | | | **Females (n=2,393)** | | | | |  |
| --- | --- | --- | --- | --- | --- | --- | --- | --- | --- | --- | --- | --- | --- |
|  | Model 1 | Model 2 | Model 3 | Model 4 | Model 5 | | Model 6 | | Model 7 | | Model 8 | Model 9 | |
|  | *OR*  *(95% CI)^a^* | *OR*  *(95% CI)^b^* | *OR*  *(95% CI)^c^* | *OR*  *(95% CI)^a^* | *OR*  *(95% CI)^b^* | | *OR*  *(95% CI)^c^* | | *OR*  *(95% CI)^a^* | | *OR*  *(95% CI)^b^* | *OR*  *(95% CI)^c^* | |
| **Caregiving group** |  |  |  |  |  | |  | |  | |  |  | |
|  |  |  |  |  |  | |  | |  | |  |  | |
| *Non-caregiving partners* | *Ref.* | | | *Ref.* | | | | *Ref.* | | | | |  |
|  |  |  |  |  |  |  | |  | |  | |  |  |
| *Care for partner with dementia* | 3.99**  (2.21, 7.20) | 2.70**  (1.46, 4.98) | 2.64** (1.36, 5.10) | 4.16*  (1.41, 12.28) | 3.17* (1.03, 9.72) | 2.64  (0.84, 8.29) | | 3.58**  (1.76, 7.26) | | 2.65*  (1.27, 5.53) | | 3.16** (1.45, 6.90) |  |
| *Care for partner with functional impairments* | 1.68*  (1.16, 2.45) | 1.24  (0.84, 1.82) | 1.15  (0.76, 1.75) | 1.73  (0.98, 3.07) | 1.18  (0.65, 2.15) | 1.15  (0.61, 2.17) | | 1.65  (0.99, 2.72) | | 1.33  (0.79, 2.23) | | 1.17  (0.66, 2.06) |  |
|  |  |  |  |  |  |  | |  | |  | |  |  |
| *Other types of partner care* | 1.82**  (1.28, 2.60) | 1.64** (1.14, 2.36) | 1.71*  (1.16, 2.51) | 1.36  (0.69, 2.70) | 1.37  (0.68, 2.77) | 1.39  (0.66, 2.94) | | 1.93**  (1.26, 2.91) | | 1.87** (1.21, 2.88) | | 1.93*** (1.23, 3.05) |  |

*Note.* CI: confidence interval; OR = odds ratio

^a^ = unadjusted model

^b^ = model adjusted for demographic factors (age, marital status, ethnicity, socioeconomic status, employment and education)

^c^ = model adjusted for demographic factors and health-related factors (presence of long-standing limiting illnesses, self-rated health, and baseline depressive symptoms); each caregiving measure was tested in a separate model

^*^ =*p* < 0.05, ^**^ =*p* < 0.005, ^***^ =*p* < 0.001


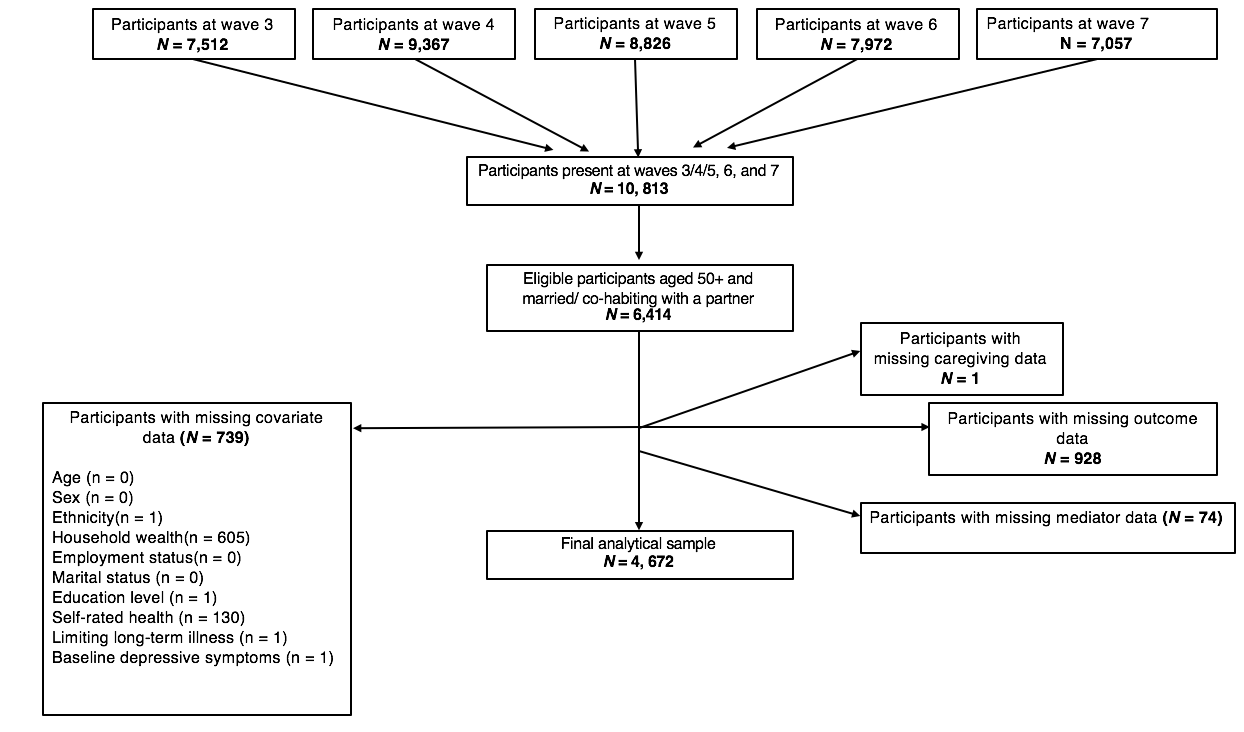


Figure 1. Participant flowchart for the analytical sample


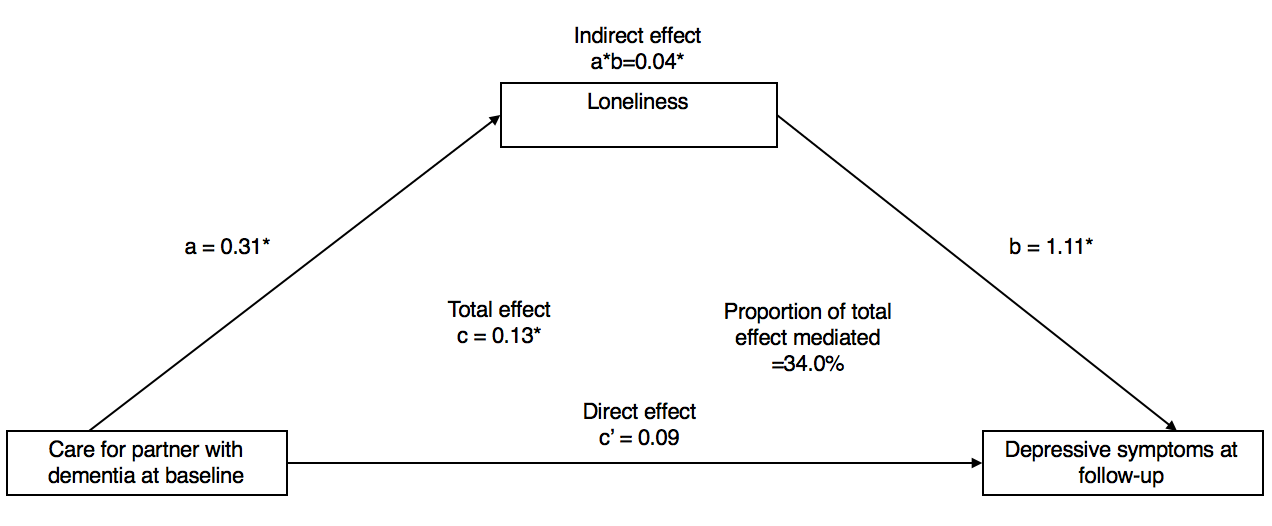


Figure 2. Mediation analysis of the association between dementia care (waves 3/4/5) on depressive symptoms (wave 7) via loneliness (wave 6) (n = 4,229)

*Note. *=p<0.05*

All models were adjusted for demographics (age, marital status, ethnicity, socioeconomic status, employment and education) and health-related (presence of long-standing limiting illnesses, self-rated health, and baseline depressive symptoms) factors. A bias-corrected bootstrap using 1000 iterations was applied to all models. Each caregiving measure was tested in a separate model.

**
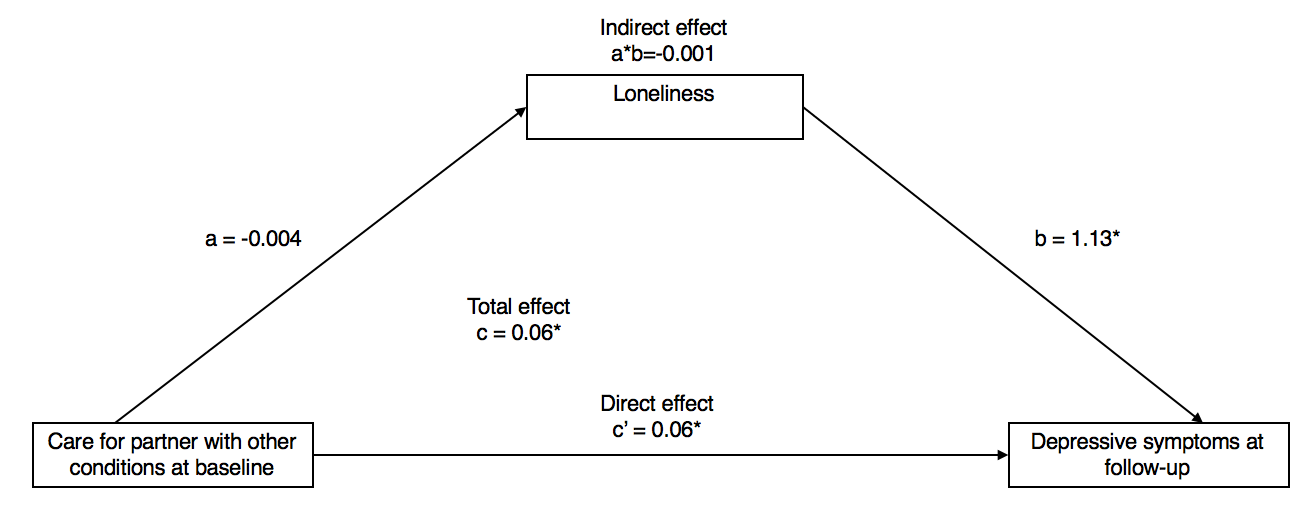
**

Figure 3. Mediation analysis of the association between ‘care for a partner with other conditions’ (waves 3/4/5) on depressive symptoms (wave 7) via loneliness (wave 6) (*n* = 4,446)

*Note. *=p<0.05*

All models were adjusted for demographics (age, marital status, ethnicity, socioeconomic status, employment and education) and health-related (presence of long-standing limiting illnesses and self-rated health) factors. A bias-corrected bootstrap with 1000 iterations was applied to all models (see supplementary materials for details).

**Supplementary material**

**Justification of covariates**

Our analyses were adjusted for baseline covariates selected based on theory and previous findings. These were derived by using their first non-missing value between waves 3 and 5. All analyses were adjusted for age, ethnicity, sex, socioeconomic status, education, employment, marital status, self-rated health and longstanding limiting illness since they are associated with the mediator and/or outcome [1-3]. Socioeconomic status was measured based on quintiles of household wealth, and wealth was measured as net wealth (sum of personal savings, investments, physical wealth and housing wealth after removal of the mortgage) minus financial debt [4]. Marital status and self-reported employment status were dichotomised. Formal education was classified into five groups (none; up to O level; A level/equivalent; higher than A level, but lower than a degree; university degree or higher).

Self-rated health was used as a proxy for health status assessed by asking participants to rate their general health on a 5-point scale ranging from poor to excellent [5]. Participants were classified as having a longstanding limiting illness if they reported the presence of any illness that limited their daily activities. The self-reported limiting longstanding illness reflects the extent to which participants feel their daily activities are limited by the presence of illness. This was assessed with two questions: (1) ‘Do you have any longstanding illness, disability or infirmity? By longstanding I mean anything that has troubled you over a period of time or that is likely to affect you over a period of time.’ Those who responded yes were asked: (2) ‘Does this illness or disability limit your activities in any way?’ Affirmation of a longstanding illness and any form of limitation classified the participant as having a limiting longstanding illness. The presence of limiting longstanding limiting illness was coded in our analyses as no, versus yes and limiting. Analyses were also adjusted for baseline depressive symptoms in order to ensure that the measured outcome was indeed the effect of caregiving rather than the presence of pre-existing symptoms of depression.

**References**

[1] Akhtar-Danesh N, Landeen J. Relation between depression and sociodemographic factors. Int J Ment Health Syst. 2007; 1: 4. https://doi:10.1186/1752-4458-1-4

[2] Victor C, Scambler S, Bowling A, Bond J. The prevalence of, and risk factors for, loneliness in later life: a survey of older people in Great Britain. Ageing Soc. 2005; 25: 357-375. https://doi:10.1017/S0144686X04003332

[3] Skarupski KA, de Leon CFM, Bienias JL, Barnes LL, Everson-Rose SA, Wilson RS, et al. Black-white differences in depressive symptoms among older adults over time. J Gerontol B Psychol Sci Soc Sci. 2005; 60: 136-142. https://doi:10.1093/geronb/60.3.p136

[4] Steptoe A, Breeze E, Banks J, Nazroo J. Cohort Profile: The English Longitudinal Study of Ageing. Int J Epidemiol. 2013; 42: 1640-8. https://doi.org/10.1093/ije/dys168

[5] Moriconi PA, Nadeau L. A Cross-Sectional Study of Self-Rated Health among Older Adults: Association with Drinking Profiles and Other Determinants of Health. Curr Gerontol Geriatr Res. 2015; 2015: 352947. https://doi:10.1155/2015/352947

**Supplementary Tables and figures**

Supplementary Table S1. The 8-item version of the Center of Epidemiological Studies-Depression Scale (Radloff, 1975)

| **The 8-items of the Center of Epidemiological Studies-Depression Scale** | | **Score** |
| --- | --- | --- |
| **1** | Whether felt depressed much of the time during the past week | yes/no - 1/0 |
| **2** | Whatever felt everything they did during the past week was an effort | yes/no - 1/0 |
| **3** | Whatever felt their sleep was restless during the past week | yes/no - 1/0 |
| **4** | Whatever was happy much of the time during the past week | yes/no - 0/1 |
| **5** | Whatever felt lonely much of the time during the past week | yes/no - 1/0 |
| **6** | Whatever enjoyed life much of the time during the past week | yes/no - 0/1 |
| **7** | Whatever felt sad much of the time during the past week | yes/no - 1/0 |
| **8** | Whatever could not get going much of the time during the past week | yes/no - 1/0 |

Supplementary Table S2. STROBE Statement—Checklist of items that should be included in reports of cohort studies

| **Item Number** | | **Recommendation** |
| --- | --- | --- |
| **Title and abstract** | 1 | (*a*) Indicate the study’s design with a commonly used term in the title or the abstract |
|  |  | (*b*) Provide in the abstract an informative and balanced summary of what was done and what was found |
| **Introduction** | | |
| Background/rationale | 2 | Explain the scientific background and rationale for the investigation being reported |
| Objectives | 3 | State specific objectives, including any prespecified hypotheses |
| **Methods** | | |
| Study design | 4 | Present key elements of study design early in the paper |
| Setting | 5 | Describe the setting, locations, and relevant dates, including periods of recruitment, exposure, follow-up, and data collection |
| Participants | 6 | (*a*) Give the eligibility criteria and the sources and methods of selection of participants. Describe methods of follow-up |
|  |  | (*b*) For matched studies, give matching criteria and number of exposed and unexposed |
| Variables | 7 | Clearly define all outcomes, exposures, predictors, potential confounders, and effect modifiers. Give diagnostic criteria, if applicable |
| Data sources/ measurement | 8* | For each variable of interest, give sources of data and details of methods of assessment (measurement). Describe comparability of assessment methods if there is more than one group |
| Bias | 9 | Describe any efforts to address potential sources of bias |
| Study size | 10 | Explain how the study size was arrived at |
| Quantitative variables | 11 | Explain how quantitative variables were handled in the analyses. If applicable, describe which groupings were chosen and why |
| Statistical methods | 12 | (*a*) Describe all statistical methods, including those used to control for confounding |
|  |  | (*b*) Describe any methods used to examine subgroups and interactions |
|  |  | (*c*) Explain how missing data were addressed |
|  |  | (*d*) If applicable, explain how loss to follow-up was addressed |
|  |  | (*e*) Describe any sensitivity analyses |
| **Results** | | |
| Participants | 13* | (a) Report numbers of individuals at each stage of study—eg numbers potentially eligible, examined for eligibility, confirmed eligible, included in the study, completing follow-up, and analysed |
|  |  | (b) Give reasons for non-participation at each stage |
|  |  | (c) Consider use of a flow diagram |
| Descriptive data | 14* | (a) Give characteristics of study participants (e.g. demographic, clinical, social) and information on exposures and potential confounders |
|  |  | (b) Indicate number of participants with missing data for each variable of interest |
|  |  | (c) Summarise follow-up time (e.g., average and total amount) |
| Outcome data | 15* | Report numbers of outcome events or summary measures over time |
| Main results | 16 | (*a*) Give unadjusted estimates and, if applicable, confounder-adjusted estimates and their precision (e.g. 95% confidence interval). Make clear which confounders were adjusted for and why they were included |
|  |  | (*b*) Report category boundaries when continuous variables were categorized |
|  |  | (*c*) If relevant, consider translating estimates of relative risk into absolute risk for a meaningful time period |
| Other analyses | 17 | Report other analyses done—e.g. analyses of subgroups and interactions, and sensitivity analyses |
| **Discussion** | | |
| Key results | 18 | Summarise key results with reference to study objectives |
| Limitations | 19 | Discuss limitations of the study, taking into account sources of potential bias or imprecision. Discuss both direction and magnitude of any potential bias |
| Interpretation | 20 | Give a cautious overall interpretation of results considering objectives, limitations, multiplicity of analyses, results from similar studies, and other relevant evidence |
| Generalisability | 21 | Discuss the generalisability (external validity) of the study results |
| **Other information** | | |
| Funding | 22 | Give the source of funding and the role of the funders for the present study and, if applicable, for the original study on which the present article is based |

*Give information separately for exposed and unexposed groups.

**Note:** An Explanation and Elaboration article discusses each checklist item and gives a methodological background and published examples of transparent reporting. The STROBE checklist is best used in conjunction with this article (freely available on the Web sites of PLoS Medicine at http://www.plosmedicine.org/, Annals of Internal Medicine http://www.annals.org/, and Epidemiology at http://www.epidem.com/). Information on the STROBE Initiative is available at http://www.strobe-statement.org.

**Sensitivity Analyses**

The association between dementia care and loneliness remained statistically significant after exclusion of those with baseline depressive symptoms (*β*=0.36, 95% CI: 0.18, 0.53), and using the higher CES-D cut-off score (*β*=0.31, 95% CI: 0.15, 0.47) (figures S1 and S2). Similarly, the association between loneliness and depressive symptoms remained significant in both analyses (after exclusion of those with baseline depressive symptoms: OR = 1.20, 95% CI: 0.99, 1.42; using higher CES-D cut-off score: OR = 1.46, 95% CI: 1.22, 1.69), as did the indirect effect of dementia care on the development of depressive symptoms via loneliness (after exclusion of those with baseline depressive symptoms: *β*=0.05, Bias corrected (Bc) CI: 0.02, 0.09; using higher CES-D cut-off score: *β*=0.04, Bias corrected (Bc) CI: 0.02, 0.07). The proportion of the total effect mediated by loneliness among these caregivers remained at 34% after excluding those with baseline depressive symptoms but increased to 46% when using the higher CES-D cut-off score.

The association between caring for a partner with other conditions and loneliness remained statistically non-significant after exclusion of those with baseline depressive symptoms (*β*=0.-0.01, 95% CI: -0.08, 0.05), and using the higher CES-D cut-off score (*β*=-0.004, 95% CI: -0.066, 0.057) (figures S3 and S4). Similarly, the association between loneliness and depressive symptoms remained significant in both analyses (after exclusion of those with baseline depressive symptoms: OR = 1.24, 95% CI: 1.02, 1.46; using higher CES-D cut-off score: OR = 1.42, 95% CI: 1.21, 1.64), as did the indirect effect of dementia care on the development of depressive symptoms via loneliness (after exclusion of those with baseline depressive symptoms: *β*=-0.002, Bias corrected (Bc) CI: -0.102, 0.007; using higher CES-D cut-off score: *β*=-0.001, Bias corrected (Bc) CI: -0.007, 0.006).

Supplementary Table S3. Logistic regression models of depressive symptomatology (wave 7) on caregiving (waves 3/4/5) after exclusion of those with baseline depressive symptoms

|  | **Full sample (n=4,249)** | | | **Males (n=2,123)** | | | **Females (n=2,126)** | | |
| --- | --- | --- | --- | --- | --- | --- | --- | --- | --- |
|  | Model 1 | Model 2 | Model 3 | Model 4 | Model 5 | Model 6 | Model 7 | Model 8 | Model 9 |
|  | *OR*  *(95% CI)^a^* | *OR*  *(95% CI)^b^* | *OR*  *(95% CI)^c^* | *OR*  *(95% CI)^a^* | *OR*  *(95% CI)^b^* | *OR*  *(95% CI)^c^* | *OR*  *(95% CI)^a^* | *OR*  *(95% CI)^b^* | *OR*  *(95% CI)^c^* |
| **Caregiving group** |  |  |  |  |  |  |  |  |  |
| *Non-caregiving partners* | *Ref.* | | | *Ref.* | | | *Ref.* | | |
| *Care for partner with dementia* | 4.57*** (2.32, 9.00) | 3.10**(1.54, 6.23) | 3.32** (1.61, 6.85) | 8.79***(2.66, 29.12) | 6.00** (1.74, 20.72) | 4.88* (1.38, 17.22) | 3.14* (1.36, 7.25) | 2.46* (1.03, 5.86) | 2.92* (1.21, 7.09) |
| *Care for partner with functional impairments* | 1.80* (1.17, 2.75) | 1.40 (0.91, 2.18) | 1.23 (0.78, 1.94) | 2.14* (1.18, 3.88) | 1.44 (0.78, 2.68) | 1.25 (0.65, 2.41) | 1.54 (0.83, 2.83) | 1.39 (0.74, 2.60) | 1.20 (0.62, 2.31) |
| *Other types of partner care* | 1.97** (1.32, 2.95) | 1.77* (1.17, 2.67) | 1.86** (1.21, 2.84) | 1.27 (0.57, 2.83) | 1.26 (0.56, 2.84) | 1.30 (0.57, 3.01) | 2.24** (1.39, 3.60) | 2.20** (1.35, 3. 60) | 2.30** (1.39, 3.81) |

*Note.* CI: confidence interval; OR = odds ratio.

^a^ = unadjusted model

^b^ = model adjusted for demographic factors (age, marital status, ethnicity, socioeconomic status, employment and education)

^c^ = model adjusted for demographic factors and health-related factors (presence of long-standing limiting illnesses, and self-rated health); each caregiving measure was tested in a separate model

^*^ *p* < 0.05, ^**^ *p* < 0.005, ^***^ *p* < 0.001

Supplementary material Table S4. Logistic regression models of depressive symptomatology (wave 7) on caregiving (waves 3/4/5) using the original CES-D scale

|  | **Full sample (n=4,672)** | | | **Males (n=2,279)** | | | **Females (n=2,393)** | | |
| --- | --- | --- | --- | --- | --- | --- | --- | --- | --- |
|  | Model 1 | Model 2 | Model 3 | Model 4 | Model 5 | Model 6 | Model 7 | Model 8 | Model 9 |
|  | *OR*  *(95% CI)^a^* | *OR*  *(95% CI)^b^* | *OR*  *(95% CI)^c^* | *OR*  *(95% CI)^a^* | *OR*  *(95% CI)^b^* | *OR*  *(95% CI)^c^* | *OR*  *(95% CI)^a^* | *OR*  *(95% CI)^b^* | *OR*  *(95% CI)^c^* |
| **Caregiving group** |  |  |  |  |  |  |  |  |  |
| *Non-caregiving partners* | *Ref.* | | | *Ref.* | | | *Ref.* | | |
| *Care for partner with dementia* | 3.87*** (2.03, 7.39) | 2.52* (1.29, 4.95) | 2.46* (1.19, 5.06) | 5.19* (1.63, 16.50) | 4.05* (1.21, 13.49) | 3.00  (0.87, 10.35) | 3.06* (1.41, 6.67) | 2.21  (0.98, 4.99) | 2.49* (1.03, 6.02) |
| *Care for partner with functional impairments* | 2.11*** (1.41, 3.17) | 1.54* (1.01, 2.35) | 1.50  (0.95, 2.36) | 2.28* (1.21, 4.30) | 1.59  (0.82, 3.08) | 1.64  (0.82, 3.26) | 2.01* (1.12, 3.43) | 1.57  (0.90, 2.73) | 1.42  (0.78, 2.60) |
| *Other types of partner care* | 1.68* (1.10, 2.55) | 1.45  (0.94, 2.24) | 1.52  (0.96, 2.40) | 1.56  (0.70, 3.47) | 1.57  (0.69, 3.56) | 1.62  (0.68, 3.88) | 1.57  (0.96, 2.58) | 1.51  (0.90, 2.51) | 1.55  (0.91, 2.65) |

*Note.* CI: confidence interval; OR = odds ratio.

^a^ = unadjusted model

^b^ = model adjusted for demographic factors (age, marital status, ethnicity, socioeconomic status, employment and education)

^c^ = model adjusted for demographic factors and health-related factors (presence of longstanding limiting illnesses, self-rated health, and baseline depressive symptoms); each caregiving measure was tested in a separate model.

^*^ *p* < 0.05, ^**^ *p* < 0.005, ^***^ *p* < 0.001


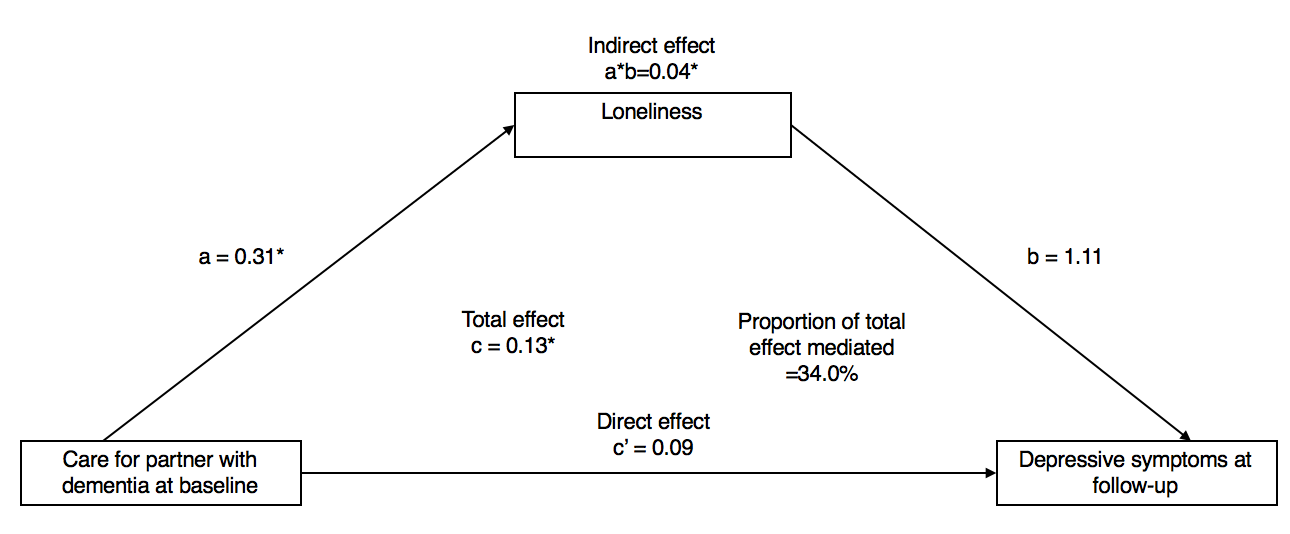


Figure S1. Mediation analysis of the association between dementia care (waves 3/4/5) on depressive symptoms (wave 7) via loneliness (wave 6) after exclusion of those with baseline depressive symptoms (n = 3, 921).

*Note. *=p<0.05*

All models were adjusted for demographics (age, marital status, ethnicity, socioeconomic status, employment and education) and health-related (presence of longstanding limiting illnesses, and self-rated health) factors. A bias-corrected bootstrap using 1000 iterations was applied to all models. Each caregiving measure was tested in a separate model.


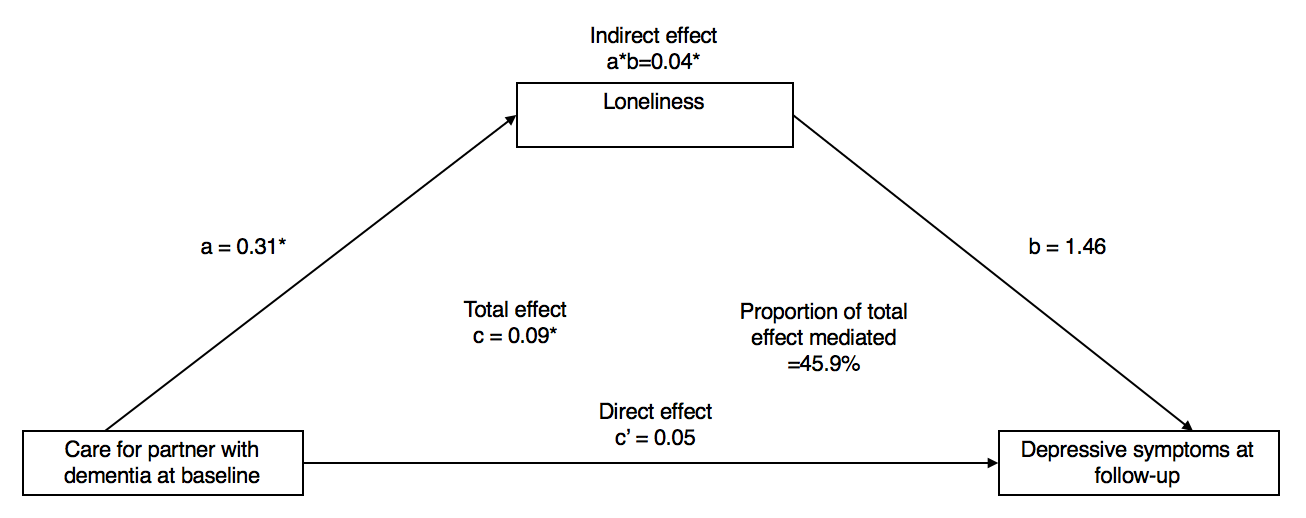


Figure S2. Mediation analysis of the association between dementia care (waves 3/4/5) on depressive symptoms (wave 7) via loneliness (wave 6), using original CES-D scale (waves 3/4/5) (n = 4,229)

*Note. *=p<0.05*

All models were adjusted for demographics (age, marital status, ethnicity, socioeconomic status, employment and education) and health-related (presence of longstanding limiting illnesses and self-rated health) factors. A bias-corrected bootstrap using 1000 iterations was applied to all models.


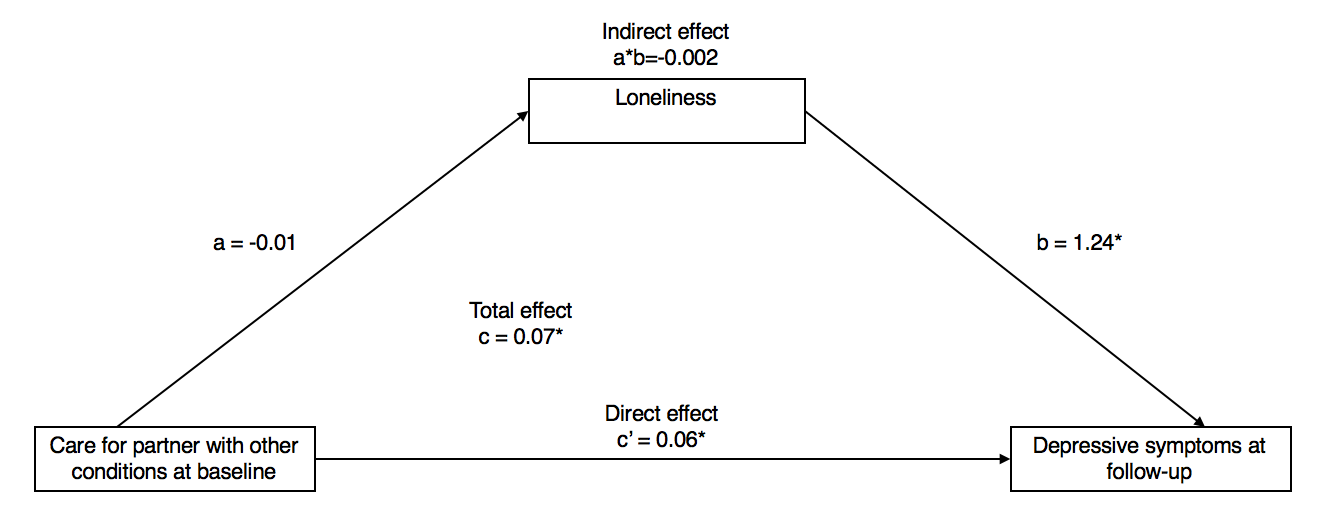


Figure S3. Mediation analysis of the association between ‘care for a partner with other conditions’ (waves 3/4/5) on depressive symptoms (wave 7) via loneliness (wave 6) (n = 4,052) after excluding those with baseline depressive symptoms (waves 3/4/5)

*Note. *=p<0.05*

All models were adjusted for demographics (age, marital status, ethnicity, socioeconomic status, employment and education) and health-related (presence of longstanding limiting illnesses and self-rated health) factors. A bias-corrected bootstrap with 1000 iterations was applied to all models.


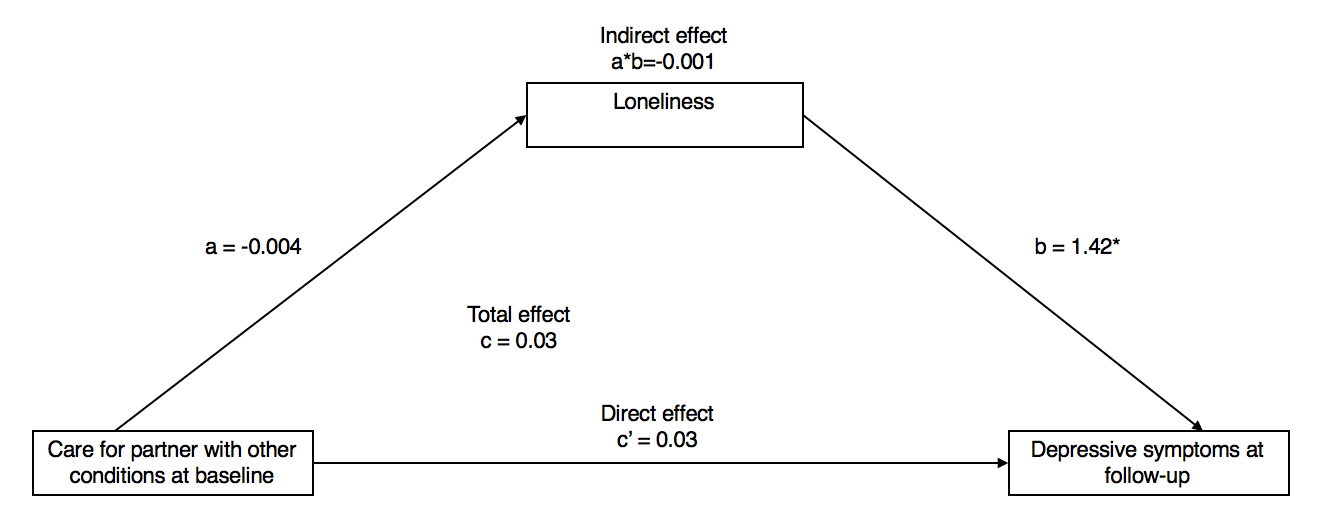


Figure S4. Mediation analysis of the association between ‘care for a partner with other conditions’ (waves 3/4/5) on depressive symptoms (wave 7) via loneliness (wave 6) (n = 4,446) using original CES-D scale (waves 3/4/5)

*Note. *=p<0.05*

All models were adjusted for demographics (age, marital status, ethnicity, socioeconomic status, employment and education) and health-related (presence of longstanding limiting illnesses, self-rated health, and baseline depression) factors. A bias-corrected bootstrap with 1000 iterations was applied to all models.
